# Australian Anaesthetist Honoured to Lead the *‘André van Zundert Research and Education Centre’* at China’s Renowned Shenzhen Brain Science and Technology Industry Innovation Center

**DOI:** 10.3390/healthcare14131860

**Published:** 2026-06-25

**Authors:** Stephen Paul Gatt

**Affiliations:** 1 Anaesthesia, Critical Care & Emergency Medicine, University of New South Wales, Sydney 2033, Australia; gattsp@gmail.com; 2 Anesthesiology and Intensive Care, Udayana University, Denpasar 80361, Bali, Indonesia

## 1. The Intersection of Clinical Safety and Advanced Research Systems: A Global Recognition of Clinical Excellence by the World’s Premier Research Body

Modern perioperative medicine stands at a critical intersection, where clinical safety frameworks must dynamically integrate with advanced biomedical technology to meet evolving global healthcare demands. Historically, clinical advancements in anaesthesiology were driven primarily by isolated bedside observations; however, modern perioperative excellence depends heavily on robust institutional structures that systematically bridge gaps among basic laboratory science, advanced engineering, and clinical translation. The continuous evolution of global healthcare delivery requires a structural transition away from fragmented clinical protocols toward comprehensive, systems-based safety paradigms. This critical evolutionary leap has been punctuated by the contributions of visionary academic clinicians who possess the rare ability to connect bench-top research directly with bedside safety. 

**Figure 1:**
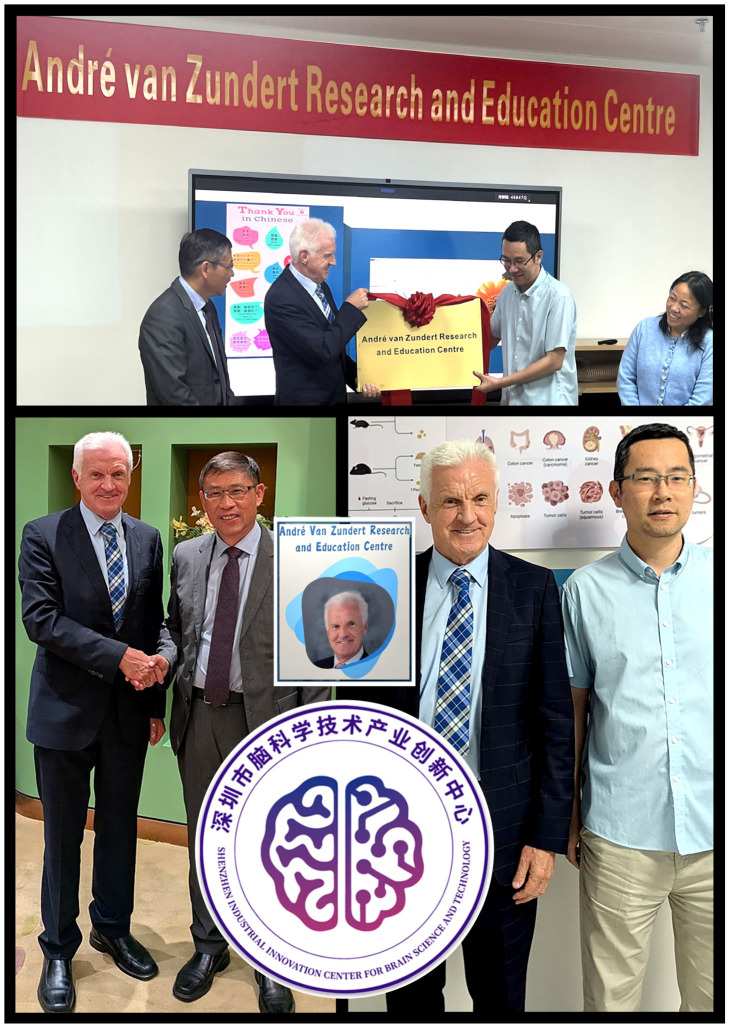
Photograph of the inaugural opening of the ‘*André van Zundert Research and Education Centre*’ at the Shenzhen Industrial Innovation Center for Brain Science and Technology on 20 April 2026. The top panel shows Prof. André van Zundert (the newly appointed chairperson) and Prof. Fuxiang He (Former First-Class Inspector of the General Office, Ministry of Science and Technology, PRC, former Counsellor of Science and Technology, Chinese Mission to the United Nations, Chinese Embassy to Denmark; current Strategic Advisor at the Shenzhen Institutes of Advanced Technology, Chinese Academy of Sciences); Prof. André van Zundert (the newly appointed chairperson); Prof. Tianwen Huang (Deputy Director of the Brain Innovation Center) and Prof. Jing He (Nanjing University of Posts and Telecommunications). The bottom panel shows Prof. Fuxiang He (**left**) and Prof. Tianwen Huang (**right**) with André van Zundert.

On 20 April 2026, a structural milestone in international medical collaboration was reached with the inauguration of the ‘*André van Zundert Research and Education Centre*’ (Figure 1), followed by a special commemorative symposium at the Shenzhen Brain Science and Technology Industry Innovation Center. Operated under the auspices of the Shenzhen Institutes of Advanced Technology (SIAT) within the Chinese Academy of Sciences—consistently recognised as a leading global research organisation in institutional impact and innovation—this Brain Innovation Center serves as the premier scientific–industrial transformation platform specialised for neuroscience research in China. For this prestigious institution to dedicate a research facility in Shenzhen, Guangdong Province, to an Australian-based anaesthetist is an exceedingly rare honour for a foreign physician and an even rarer distinction for the field of anaesthesiology. This landmark establishment is a profound testament to the universal, transcontinental impact of Professor van Zundert’s scientific legacy, clinical acumen, and lifelong contributions to patient safety and clinical innovation. By situating an international perioperative and anaesthesia-focused facility directly within a world-class neuroscience and advanced technology ecosystem, the centre establishes a revolutionary precedent. It shifts the traditional, isolated educational model toward a highly integrated, fluid system where advanced engineering, neuroinformatics, molecular pharmacology, and clinical practice informing and enriching one another in real time. 

This newly established centre represents a strategic international platform designed to operationalise the “Big Five” conceptual pillars of academic anaesthesia: teaching, training, testing, quality, and research. Concurrently, this editorial outline the transformative career of Professor André van Zundert, highlighting his future role in advancing global healthcare standards through innovations in airway management, obstetric and regional anaesthesia, and pharmacological research into the mechanisms of action of general anaesthetics and their reversal agents. Ultimately, this text examines how integrating advanced translational research platforms with modern educational paradigms—such as high-fidelity simulation, technology-driven quality audits, and validated knowledge-crowdsourcing frameworks—can completely redefine patient safety, optimise pharmacological delivery, and build sustainable international capacity for the next generation of anaesthesiologists. 

## 2. Conceptual Foundation: Operationalising the “Big Five” Paradigm

To understand the international trajectory of the new centre, its curriculum and research programmes must be examined through established academic frameworks. The structural blueprint of the centre is explicitly modelled on the “Big Five” core pillars of academic anaesthesia originally articulated in the British Journal of Anaesthesia [1]: (1) Teaching and Learning (Education): Moving beyond traditional passive lectures to active, learner-centred paedagogy. (2) Training (Skill Acquisition and Protocol Development): Emphasising objective technical competence and behavioural performance. (3) Testing (Systems, Theories, and Equipment): Rigorous, evidence-based assessment of clinical tools and workflows prior to widespread deployment. (4) Quality (Innovation and Excellence of Care): Implementing systematic clinical audits, human factors engineering, and ‘Safety-II’ resilience engineering. (5) Ongoing Research (Basic and Clinical): Fostering continuous inquiry into both molecular mechanisms and clinical effectiveness.

Historically applied within the awarded ‘Centre for Excellence and Innovation in Anaesthesia’ (CEIA), personally conceptualised and established by Professor van Zundert at the Royal Brisbane and Women’s Hospital and the University of Queensland, Brisbane, Australia, this framework has demonstrated that clinical departments can be systematically transformed into high-output research powerhouses with international reach. The Shenzhen Centre scales this methodology by embedding these five elements directly into an industrial-scale technology incubator, providing a unique global model for training and research.

The International Research Center for Pain Management and Anesthesiology in Shenzhen, led by Professor André van Zundert as the Chairperson of the Academic Committee, brings together renowned scholars from home and abroad to form a highly skilled professional academic team. It focuses on areas such as brain research involving anaesthesia, knowledge mapping in pain management and anaesthesia, anaesthesia robots, traditional Chinese medicine, anaesthesia innovation, mechanisms of action and reversal agents for general anaesthetics, artificial intelligence and anaesthesia, and aims to conduct cutting-edge research and drive clinical transformation in these fields.

Professor van Zundert has maintained an extraordinary clinical workload, having personally administered over 55,000 anaesthetics throughout his career and was frequently assigned the most difficult and high-risk patients, consistently guiding them to safety through his expert clinical acumen. Professor van Zundert’s career—spanning several decades across Europe and Australia—is defined by a relentless pursuit of clinical excellence and quality care. Currently serving as the Professor and Chairman of the Discipline of Anaesthesiology at the Royal Brisbane and Women’s Hospital (RBWH) and the University of Queensland, his work has consistently moved research from concept into real-world application. This global recognition follows a series of recent prestigious accolades, including the 2024 AMA Excellence in Healthcare Award (Australian Medical Association), the 2024 Leonard Travers Professorship, the 2024 ANZCA Medal and the 2026 Robin Smallwood Award (Australian and New Zealand College of Anaesthetists), and the 2025 Carl Koller Gold Medal (European Society of Regional Anaesthesia and Pain Therapy), alongside his listing in the World’s Top 2% Scientists, acknowledging his profound influence on the healthcare landscape [2,3,4,5].

## 3. Leadership in Scholarly Dissemination: The Healthcare Journal

Central to Professor van Zundert’s mission to elevate medical standards is his leadership as Editor-in-Chief of the Clinical Care section of the journal *Healthcare*. This international, peer-reviewed, open-access journal serves as a vital platform for disseminating high-impact research on healthcare systems, industry, and technology. Prof van Zundert plays a pivotal role in promoting clinical excellence. He fosters a rigorous academic environment that prioritises translating theoretical research into practical, life-saving bedside applications. The journal *Healthcare* has become a leading voice in promoting patient safety and perioperative innovation, ensuring that groundbreaking findings reach a global audience of practitioners and policymakers.

- **Pioneering Safety in Obstetric and Regional Anaesthesia**

Professor van Zundert’s scholarly output includes some 150 scientific articles dedicated specifically to the safety of obstetric anaesthesia [6,7,8,9,10]. The formula he proposed for safe and effective pain relief during childbirth is now a staple of clinical practice worldwide. His work has redefined how clinicians approach pain relief during childbirth, focusing on evidence-based protocols that minimise risk to both mother and neonate.

In the broader field of regional anaesthesia, in which he has authored another 150 articles [11], he co-authored the most-cited article of all time in the discipline [12]. As one of the founders of the ‘Combined Spinal-Epidural Technique’, he provided clinicians with a versatile tool that balances rapid onset with long-term pain management [13,14]. Perhaps his most significant clinical contribution is the introduction of the ‘*Segmental Thoracic Spinal Anaesthesia*’ technique [15]. This innovation represents a life-saving intervention for high-risk patients with severe comorbidities, such as extreme pulmonary compromise. It has facilitated successful surgeries for tens of thousands of patients who likely would not have survived the physiological stress of general anaesthesia.

- **From Mirrors to Monitors: The Revolution in Airway Management**

Recognised as one of the ‘Top Ten World Experts in Airway Management’, Professor van Zundert has produced approximately 150 airway-related scientific publications, including world guidelines. He was a foundational figure and one of the early promoters of videolaryngoscopy, moving the speciality ‘from mirrors to monitors’ [16,17,18,19,20,21,22,23,24,25,26,27].

By championing the transition from ‘blind’ clinical techniques to ‘vision-guided’ precision, this ‘*King of Airways*’ has achieved a monumental leap in patient safety. His clinical vision further led to the introduction of vision-incorporated third-generation videolaryngeal mask airways (VLMAs) into routine practice, providing clinicians with unprecedented control and positioning accuracy during difficult airway management [28,29,30,31,32,33,34,35,36,37].

- **Unlocking the Mechanisms of General Anaesthesia**

Beyond clinical tools, Professor van Zundert is at the forefront of unravelling the molecular mechanisms of general anaesthesia through his appointments at the Queensland Brain Institute (QBI), The University of Queensland, and the Queensland University of Technology in Brisbane, Australia: (1) Presynaptic Discoveries: His research team at QBI discovered that general anaesthetics significantly affect the presynaptic transmitter-release machinery, a finding that fundamentally alters our understanding of induced unconsciousness [38]. (2) Next-Generation Pharmacology: Currently, a new propofol-type anaesthetic is being tested (including research into Ciprofol) at the van Swinderen Lab at QBI that aims to replace traditional propofol. This new agent is designed to be safer and more potent, with significantly fewer side effects. (3) Reversal Agents: Crucially, his team is researching new reversal agents that counteract general anaesthetics, promising to re-volutionise the recovery phase by enabling patients to wake up more rapidly and with fewer cognitive complications [39].

- **Mentorship, Education, Simulation and the ‘Safety-II’ Paradigm**

Professor van Zundert’s impact is equally profound in the realm of medical education and simulation: (1) High-End Simulation Manikins: By utilising advanced, physiologically responsive tetherless manikins that replicate real-time pharmacological and clinical crises, training programmes can build critical clinical muscle memory. These high-end systems simulate reactive pupils, complex airway swelling, tongue oedema, laryngospasm, and changes in compliance, allowing trainees to manage catastrophic events safely. (2) Simulation Accuracy and Pathophysiology: The amalgamation of machine learning (ML) and high-fidelity simulators allows educators to mimic specific complex, low-frequency, high-acuity cases (e.g., peripartum cardiomyopathy, anaphylaxis, malignant hyperthermia, or total spinal anaesthesia) to test drug and intervention responses before the actual bedside procedure. (3) Immersive Reality and Planning: Using 3D-reconstructed CT images and Virtual Reality (VR), combined with physical haptic simulators, enables clinicians to plan and practice airway management in severely compromised anatomy.

Having supervised over 50 successful PhD graduates—eleven of whom have become full professors—he prioritises a ‘theatre-as-classroom’ model that emphasises hands-on training and learner-centred feedback. Furthermore, his advocacy for ‘Safety-II’ and ‘Just Culture’ principles has shifted the focus of incident investigation from individual blame to systemic resilience, ensuring that healthcare systems learn as much from success as they do from failure.

- **Institutional Transformation and Research Innovation at RBWH, Australia**

Upon his appointment, Professor van Zundert was tasked with establishing a robust academic department of anaesthesia at the Royal Brisbane and Women’s Hospital (RBWH) and the University of Queensland (UQ). Since his arrival in Brisbane approximately 13 years ago, he has successfully transformed the Department of Anaesthesia at the Royal Brisbane and Women’s Hospital into a premier research and training powerhouse, leaving an indelible mark on the international clinical and academic forum.

In 2024, UQ awarded him its highest diploma, a ‘*Doctorate in Medicine by Research*’, in recognition of his contributions to airway management [40]. At the same time, it underpins André’s lifelong adherence to study and his belief that a ‘good teacher is also a good student’. He personally established, directed, and funded the UQ-awarded ‘*Centre for Excellence and Innovation in Anaesthesia* (CEIA)’, which has since produced dozens of highly competent anaesthesiologists with advanced clinical skills [41]. The British Journal of Anaesthesia published this CEIA concept as the way forward for anaesthesia education, based on the following five pillars: teaching, training, testing, quality care, and research [1,42,43].

- **Humanitarian and Charitable Contributions and International Leadership**
  - Philanthropic Leadership: Beyond his academic and clinical roles, Professor van Zundert is deeply committed to humanitarian service, frequently dedicating his time to feeding and clothing the homeless and vulnerable populations at Emmanuel City Mission and the Order of Malta in Brisbane, Australia. Furthermore, he is a Knight of the Equestrian Order of the Holy Sepulchre of Jerusalem.
  - Recognition of Character: His conferment as a Commander of the Order of the Crown of the Kingdom of Belgium (2024) specifically cited his ‘contributions to several areas of humanitarian, charitable and philanthropic endeavours’ alongside his medical achievements [44].
  - Distinguished Military Career: A retired Medical Lieutenant Colonel, Professor van Zundert served for 30 years as a reservist in the Belgian military. His service included spearheading medical support for a major peacekeeping mission in Benin, Africa.
  - Global Capacity Building: On the international stage, he single-handedly established an anaesthesia department in Sint Maarten (Caribbean), where he successfully trained a complete anaesthesia and pain management team.
  - European Leadership: Before moving to Australia, he was already a foundational figure in Europe, serving as the Secretary General and later, as the President of the European Society of Regional Anaesthesia and Pain Therapy (ESRA) and as the founding father and chair of the examining board for the European Diploma of Regional Anaesthesia (EDRA).
- **Bridging Global Clinical Frameworks and Advanced Technology: The Future of Research, Education, and Safety in Perioperative Medicine, Anaesthesiology and Pain Medicine**

○ Anaesthesiologists have unique safety tasks for patients undergoing anaesthesia, perioperative care, intensive care medicine, pain therapy and emergency medicine. Although the overall improvement in mortality risk from complications and adverse events over the years is obvious [45], nowadays, anaesthesiologists need to take care of patients who are older, more obese, with more comorbidities, undergoing more aggressive surgical interventions, using better monitoring and equipment, and newer drugs, there is still room for improvement. Better training of future anaesthesiologists, high-fidelity simulation modelling, advanced research, and translational innovation are needed to further improve our service to patients. Future research (Table 1) should explore additional strategies to improve compliance with targeted multidisciplinary education and training initiatives, enhance awareness, and optimise the monitoring and management of at-risk patients, with a focus on prevention, e.g., in difficult airway management. The ‘*André van Zundert Research and Education Centre’* is committed to the global advancement of the speciality, to improving systemic safety and efficiency in anaesthesia care for patients, and to training highly qualified doctors.
○ The positioning of the new centre within a specialised neuroscience and advanced technology hub enables it to lead several critical frontiers in perioperative science: e.g.,:
  **(1)**  ***Advanced Airway Technology and Vision-Guided Interventions***
    The transition from traditional, blind airway interventions to vision-guided techniques remains one of the most effective safety vectors in modern anaesthesia. Future directions require moving beyond standard videolaryngoscopy into the integration of artificial intelligence and automated imaging. The centre’s research focus includes the development, testing, and clinical validation of next-generation videolaryngeal mask airways (VLMAs) and smart-sensor devices. This continuous optimisation aims to virtually eliminate unrecognised oesophageal intubation and minimise airway trauma across diverse patient populations.
  **(2)**  ***Presynaptic Mechanisms of General Anaesthetics***
    A critical gap in modern pharmacology is the precise understanding of how general anaesthetics alter synaptic transmission at the molecular level. Building upon basic neuroscience collaborations—including insights from the Queensland Brain Institute—researchers at the centre are investigating how anaesthetic agents interact with the presynaptic transmitter-release machinery. Unlocking these specific pathways is essential for designing targeted agents that induce predictable unconsciousness while minimising systemic toxicity.
  **(3)**  ***Next-Generation Pharmacotherapy: Safety and Targeted Reversal***
    The clinical reliance on traditional intravenous anaesthetics, such as propofol, is frequently limited by predictable side effects, including injection pain, respiratory depression, post-induction hypotension, and cardiovascular desaturation. The primary objective of the centre is the evaluation and testing of new propofol-type compounds (including advanced iterations of agents like Ciprofol).
      These next-generation alternatives possess a significantly higher therapeutic index (TI), widening the margin between the therapeutic and toxic doses. Furthermore, the centre’s research programmes prioritise developing targeted reversal agents for general anaesthetics. Achieving rapid, pharmacologically driven recovery will minimise postoperative cognitive dysfunction (POCD) and accelerate discharge pathways, particularly within fast-track ambulatory surgery systems.
  **(4)**  ***Regional and Neuraxial Innovations for High-Risk Sub-populations***
    As global demographics shift toward an ageing, increasingly comorbid surgical population, the reliance on traditional general anaesthesia must be re-evaluated. The optimisation of non-pharmacological and localised techniques—such as segmental thoracic spinal anaesthesia—has demonstrated a profound capacity to save lives in patients with severe respiratory or cardiovascular compromise who might not survive standard general endotracheal anaesthesia. The centre will serve as an international research node for mapping functional anatomy and defining low-dose combinations of local anaesthetics to expand the safety boundaries of regional and obstetric anaesthesia.

- **Modernising Educational Paradigms: High-Fidelity Simulation and Digital Knowledge Networks**

To successfully prepare the next generation of anaesthesiologists, medical education must discard unstructured clinical exposure in favour of rigorous, reproducible simulation-based training.

**(1)**  High-Fidelity Simulation as a Safety Core

The centre is designed to utilise advanced simulation-based education to mimic complex crisis resource management (CRM) scenarios. By testing new medical devices and validating clinical workflows within controlled, simulated environments before clinical introduction, the centre links educational research directly to patient safety outcomes. This structured methodology ensures that technical skills are acquired without exposing patients to the inherent learning curves of novel interventions.

**(2)**  Validated Crowdsourcing and Global Knowledge Repositories

A persistent challenge in global healthcare is the fragmentation and variable quality of online medical information. To address this, the centre incorporates validated, open access, and crowdsourced repositories of anaesthesia information, building upon the published foundational methodology [42,43]. This structured model of collective intelligence—widely recognised and analysed by major international institutions, including Johns Hopkins University—uses expert peer-matching and self-regulating frameworks to synthesise complex clinical data into actionable, peer-reviewed clinical summaries. The centre will expand this digital architecture, providing an international platform to host open access, evidence-based medical knowledge that mitigates reliance on unvalidated internet resources.

- National and International Perspectives: What the Centre Offers to Global Healthcare

The establishment of an autonomous research and education centre focused on anaesthesiology within a premier national academy of sciences carries profound international implications (Table 2). Historically, dedicated research centres are infrequently awarded to foreign clinicians, and even less frequently to the speciality of anaesthesiology, which is often viewed as a purely supportive clinical service rather than an independent scientific discipline.

- **Conclusions: A Transformative Legacy Offering a Balanced Matrix for the Future of Care in Anaesthesia**

The establishment of the ‘*André van Zundert Education and Research Centre*’ in Shenzhen—located in Guangdong Province, often nicknamed ‘China’s Silicon Valley’ for its rapid transformation into a global technology and innovation hub—is a testament to a career that has achieved the rare feat of being both highly specialised and globally applicable. With over 550 scientific articles, 80+ book chapters, and 8 major textbooks, Professor van Zundert’s data-driven approach has undeniably made surgery safer for millions. His work beautifully bridges the gap among theoretical excellence, up-to-date simulation and training, improvements in the quality of care, and life-saving governance.

The new centre at the Brain Science and Technology Industry Innovation Center of SIAT is structured to transcend its local environment. By balancing technological innovation with a rigorous commitment to the “Big Five” academic pillars, the centre shifts the discipline’s focus from reactive error avoidance to proactive, systems-level resilience. Through its dedicated lines of research in molecular pharmacology, vision-guided airway management, and open-access digital education networks, the centre provides a scalable blueprint for the future of global perioperative medicine. Ultimately, its success will be measured not by institutional prestige but by its practical, data-driven contributions to making anaesthesia care safer, more effective, and accessible to patients worldwide.

By championing advanced research into AI-driven haemodynamic forecasting, computer-vision airway modelling, and continuous tracking via the ‘Perioperative Human Cloud,’ the centre is poised to redefine how perioperative medicine adapts to an increasingly complex, ageing, and comorbid patient population. Furthermore, this legacy strongly reinforces the clinical environment by integrating high-end, high-fidelity simulation manikins. By allowing future anaesthesiologists to master complex, low-frequency clinical crises and difficult airway scenarios in a zero-risk environment, simulation becomes the ultimate vehicle for translating Safety-II principles into daily practice. Coupled with the ‘Wiki-Anaesthesia’ open-source vision, the centre ensures that medical education evolves dynamically in step with the rapid pace of scientific advancement. Crucially, the centre arrives at a pivotal inflexion point for the speciality, serving as an international launchpad for the next-generation clinical and educational paradigms. Having supervised more than 50 PhD candidates, van Zundert considers his true legacy to be this next generation of clinical leaders and scientists who will spearhead these transformations. As the Editor-in-Chief of Healthcare—Section Clinical Care, he continues to provide the global leadership necessary to push the boundaries of medical innovation. Armed with these promising assets, the new centre is uniquely equipped to play a major, defining role at the World Forum of Anaesthesia, ensuring that the legacy of safety he has built continues to safeguard patients for generations to come.

## Figures and Tables

**Table 1:** Future directions in anaesthesiology—a non-exhaustive list of suggestions for improvement.

Category	Future Research and Improvements
**Patient Safety and Monitoring**	Airway Management: Development of computer vision to predict difficult intubation by analysing facial features and anatomical distances with sub-millimetre precision. Haemodynamics: Integration of AI to predict hypotensive episodes minutes before they occur using arterial pressure waveform analysis. Anaesthesia Depth: Closed-loop systems using EEG-guided dosing to maintain target hypnosis and reduce ‘overshoot’. Thermal Management: Intelligent monitoring of hypothermia using medical-grade wearables for real-time perioperative tracking.
**High-Fidelity Simulation and Advanced Training**	High-End Simulation Manikins: Utilising advanced, physiologically responsive tetherless manikins that replicate real-time pharmacological and clinical crises. These high-end systems simulate reactive pupils, complex airway swelling, tongue oedema, laryngospasm, and changes in compliance, allowing trainees to manage catastrophic events safely. Simulation Accuracy and Pathophysiology: Amalgamation of machine learning (ML) and high-fidelity simulators to mimic specific complex, low-frequency, high-acuity cases (e.g., peripartum cardiomyopathy, anaphylaxis, malignant hyperthermia, or total spinal anaesthesia) to test drug and intervention responses before the actual bedside procedure. Immersive Reality and Planning: Using 3D-reconstructed CT images and Virtual Reality (VR) combined with physical haptic simulators to plan and practice airway management in severely compromised anatomy.
**Remote &** **Continuous Wearable Monitoring**	The ‘Perioperative Human Cloud’: Anaesthesia safety expanding beyond operating theatres into the ‘hospital-at-home’ model where patients are monitored pre- and post-surgery using medical-grade wearables tracking ECG, oxygenation, and activity levels for prehabilitation and early detection of complications like post-discharge respiratory depression. Augmented Reality (AR) Overlays: Using AR headsets to see real-time vital signs and 3D anatomical reconstructions overlaid directly onto the patient during a procedure, maintaining ‘eyes-on-patient’ at all times.
**Perioperative Brain Health**	Cognitive Recovery: Prioritising postoperative delirium, postoperative cognitive dysfunction, cerebral oxygenation, inflammation, frailty, and depth of anaesthesia monitoring, particularly in older surgical populations using EEG-based technologies like Bispectral Index (BIS) and Density Spectral Array (DSA). Frailty Metrics: Integrating frailty and ageing indices as primary predictors of complications, delirium, disability, discharge destination, and mortality.
**AI Predictive Analytics**	Event Prediction: Machine learning (ML) models forecasting respiratory desaturation and postoperative pulmonary complications based on real-time physiologic signals (capnography/ventilatory curves). Risk Profiling: Natural language processing (NLP) extracting ‘hidden’ risk factors from unstructured clinical notes to automate ASA classification. Ideal Data Recording: Shifting from reactive to ‘predictive’ context-aware workstations that integrate multimodal data into smart alerts to eliminate alarm fatigue.
**Quality and Guidelines**	Systemic Safety (Safety-II): Moving from ‘policing mistakes’ to learning from ‘work-as-done’ through proactive tools like Failure Mode and Effects Analysis (FMEA). Dynamic Guidelines (Wiki-Anaesthesia): Transitioning to ‘two-page’ open-source knowledge modules (1 page theory, 1 page visual/graphical) to combat information overload, and utilising crowdsourced networks to update guidelines dynamically.

**Table 2:** National and international perspectives: what the centre offers to global healthcare.

Dimension	Future Offerings and Global Impact
**International Capacity Building**	Serving as a bridge between Western and Eastern academic institutions, facilitating bilateral research fellowships, PhD supervision, and academic exchanges.
**Technological Incubation**	Providing a physical environment where clinical anaesthesiologists can collaborate directly with CAS engineers and neuroscientists to design, prototype, and test novel medical devices.
**Systemic Safety Science**	Hosting global multi-centre clinical audits, applying “Safety-II” concepts to complex healthcare workflows, and establishing international guidelines for perioperative risk mitigation.
**Open Access Educational Equity**	Utilising digital infrastructure to distribute high-fidelity curricula, simulation designs, and crowdsourced knowledge repositories to low- and middle-income countries.
